# Influence of phytosomal curcumin on anthropometric indices for nonalcoholic fatty liver disease: A meta-analysis

**DOI:** 10.1097/MD.0000000000040538

**Published:** 2024-12-27

**Authors:** Nana Liu, Hongting Li

**Affiliations:** a Department of Hepatic Diseases, Chongqing Traditional Chinese Medicine Hospital, Chongqing, China; b Chongqing College of Traditional Chinese Medicine, Chongqing, China.

**Keywords:** anthropometric indices, nonalcoholic fatty liver disease, phytosomal curcumin, randomized controlled trials

## Abstract

**Background:**

Phytosomal curcumin may have some potential in improving anthropometric indices for nonalcoholic fatty liver disease, and this meta-analysis aims to study the effect of phytosomal curcumin on anthropometric indices for nonalcoholic fatty liver disease.

**Methods:**

We have searched several databases including PubMed, EMbase, Web of science, EBSCO and Cochrane library databases, and selected the randomized controlled trials (RCTs) comparing phytosomal curcumin with placebo for nonalcoholic fatty liver disease. This meta-analysis was conducted using the random-effect or fixed-effect model based on the heterogeneity.

**Results:**

Seven RCTs were included in this meta-analysis. Compared with placebo in nonalcoholic fatty liver disease, phytosomal curcumin was associated with substantially reduced body mass index (BMI, mean difference [MD] = −0.72; 95% confidence interval [CI] = −1.11 to −0.33; *P* = .0003), body fat percent (MD = −2.04; 95% CI = −3.10 to −0.98; *P* = .0002), weight (MD = −2.15; 95% CI = −3.78 to −0.53; *P* = .01) and waist circumference (MD = −2.09; 95% CI = −4.05 to −0.12; *P* = .04), but showed no influence on hip circumference (MD = −0.06; 95% CI = −0.59 to 0.47; *P* = .83) or waist/hip ratio (MD = 0; 95% CI = −0.01 to 0.01; *P* = 1).

**Conclusions:**

Phytosomal curcumin is effective to improve anthropometric indices for patients with nonalcoholic fatty liver disease.

## 1. Introduction

Nonalcoholic fatty liver disease is known as the most prevalent liver disease.^[1–4]^ It is characterized by the accumulation of neutral lipids in liver cells, such as triglycerides.^[5–7]^ The progression of nonalcoholic fatty liver disease leads to liver fibrosis, nonalcoholic steatohepatitis, cirrhosis and carcinoma.^[8]^ As the increasing prevalence in type 2 diabetes and obesity, the epidemiology of nonalcoholic fatty liver disease has been growing.^[9–11]^ Especially, nonalcoholic fatty liver disease is closely associated with diabetes, metabolic syndrome, obesity and dyslipidemia.^[12–14]^

As 1 polyphenolic component in turmeric, curcumin is first shown to have antibacterial activity.^[15]^ Animal and human studies indicate that curcumin contains hypoglycemic, wound healing, anti-inflammatory, antiviral, anticancer, and antioxidant properties.^[16–19]^ Curcumin has important capability to treat fatty liver by improving the insulin sensitivity, decreasing the inflammation and oxidative stress.^[20]^ However, it is limited by poor bioavailability, absorption, and metabolism.

In order to increase the bioavailability, curcumin in phytosome form has intermolecular bonding with phospholipid molecules, which benefits to stabilize curcumin.^[21,22]^ However, the effect of phytosomal curcumin on anthropometric indices for nonalcoholic fatty liver disease remains unclear. Several studies reported the potential of phytosomal curcumin to improve the anthropometric indices, but the results were not well established.^[8,23–25]^ This meta-analysis of randomized controlled trials (RCTs) aims to explore the effect of phytosomal curcumin vs placebo on anthropometric indices for nonalcoholic fatty liver disease.

## 2. Materials and methods

This meta-analysis was conducted according to the Preferred Reporting Items for Systematic Reviews and Meta-analysis statement and Cochrane Handbook for Systematic Reviews of Interventions (Table S1, Supplemental Digital Content, http://links.lww.com/MD/O221).^[26,27]^ No ethical approval or patient consent were required because all analyses were based on previous published studies.

### 2.1. Literature search and selection criteria

Search several databases were systematically searched by 2 independent authors from inception to December 2023 by using the keywords: “nonalcoholic fatty liver disease” AND “curcumin.” They included PubMed, EMbase, Web of science, EBSCO and the Cochrane library. The inclusion criteria were presented as follows: study design was RCT, patients were diagnosed with nonalcoholic fatty liver disease, and intervention treatments were phytosomal curcumin vs placebo. Phytosomal curcumin was defined as the curcumin in phytosome form that has intermolecular bonding with phospholipid molecules. Studies reported curcumin were excluded.

### 2.2. Data extraction and outcome measures

We extracted the baseline information including first author, number of patients, age, female, weight, body mass index and detail methods. Data were extracted independently by 2 investigators, and discrepancies were resolved by consensus. The primary outcomes were body mass index (BMI) and body fat percent. The BMI was calculated as the weight-to-height squared ratio. Secondary outcomes included weight, waist circumference, hip circumference and waist/hip ratio.

### 2.3. Assessment for risk of bias

The risk of bias tool was used to assess the quality of individual studies in accordance with the Cochrane Handbook for Systematic Reviews of Interventions,^[28]^ and the following sources of bias were considered: selection bias, performance bias, attrition bias, detection bias, reporting bias, and other potential sources of bias. The overall risk of bias for each study was evaluated and rated: low, when the risk of bias was low in all key domains; unclear, when the risk of bias was low or unclear in all key domains; and high, when the risk of bias was high in 1 or more key domains.^[29]^ Two investigators independently assessed the quality of included studies. Any discrepancy was solved by consensus.

### 2.4. Statistical analysis

Mean difference (MD) with 95% confidence interval (CI) was used to evaluate all continuous outcomes. Heterogeneity was evaluated using the *I*^2^ statistic, and *I*^2^ > 50% indicated significant heterogeneity.^[30]^ This meta-analysis was performed by using the random-effect model for significant heterogeneity, and otherwise fixed-effect model was used. Sensitivity analysis was conducted to detect the influence of a single study on the overall estimate via omitting 1 study in turn or performing the subgroup analysis. Publication bias was not assessed due to the limited number (<10) of included studies. Results with *P* < .05 were considered to have significant difference. All statistical analyses were performed using Review Manager Version 5.3 (The Cochrane Collaboration, Software Update, Oxford, UK).

## 3. Results

### 3.1. Literature search, study characteristics and quality assessment

**Figure 1.**
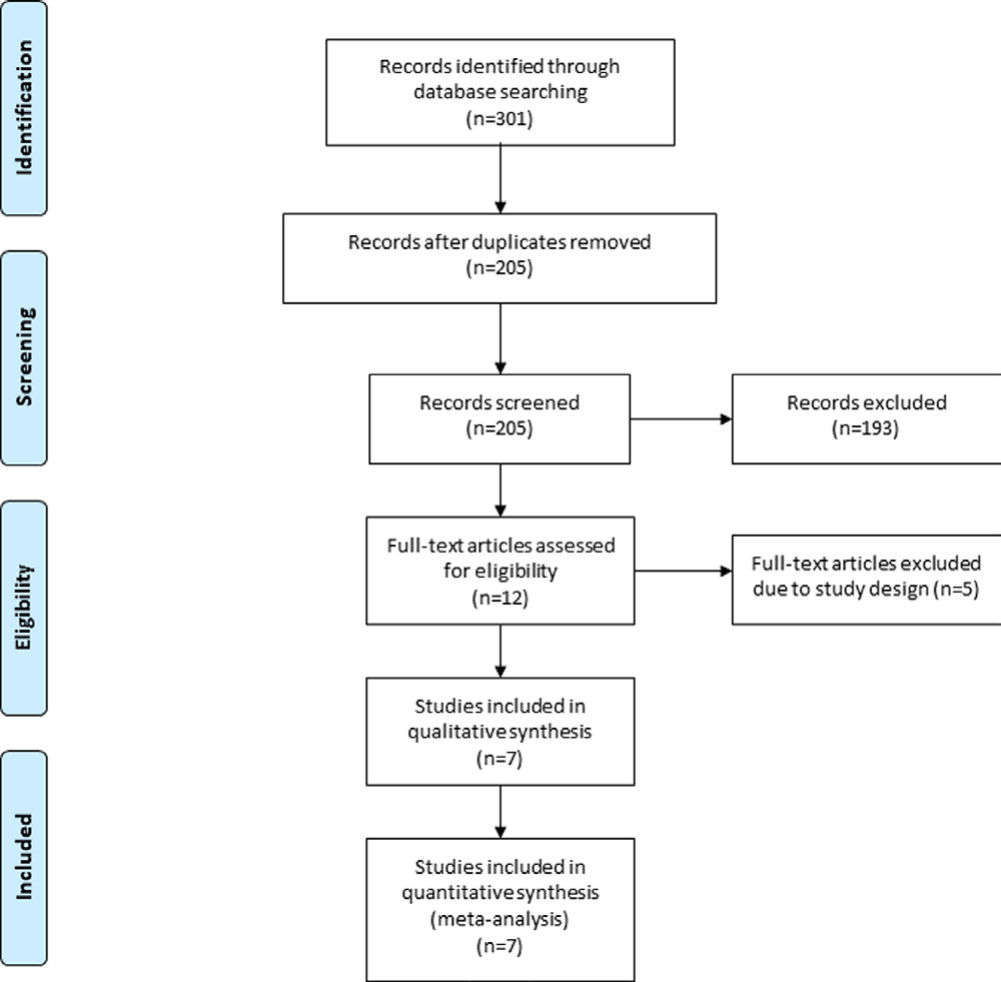
Flow diagram of study searching and selection process.

The detail flowchart of the search and selection results was presented in Figure 1. Initially, 301 potentially relevant articles were found and 7 RCTs were finally included in this meta-analysis (Table S2, Supplemental Digital Content, http://links.lww.com/MD/O221).^[8,23–25,31–33]^ The baseline characteristics of 7 included RCTs were shown in Table 1. These studies were published between 2017 and 2023, and the total sample size was 443.

**Table 1:** Characteristics of included studies.

No	Author	Curcumin group						Control group						Jada scores
		Number	Age (yr)	Female (n)	Weight (kg)	Body mass index (kg/m^2^)	Methods	Number	Age (yr)	Female (n)	Weight (kg)	Body mass index (kg/m^2^)	Methods	
1	Safari 2023	28	43.92 ± 8.74	11	82.72 ± 13.48	28.95 ± 3.24	250 mg daily phytosomal curcumin for 12 wk	28	50.35 ± 9.44	17	79.59 ± 8.81	29.28 ± 3.30	Placebo	5
2	Mirhafez 2021	22	41.2 ± 14.1	7	78.4 ± 10.2	31.1 ± 2.14	250 mg/day phytosomal curcumin for 8 wk	22	40.7 ± 11.0	9	80.0 ± 15.1	30.5 ± 4.16	Placebo	4
3	Mirhafez 2021 (2)	35	45.0 ± 11.1	16	85.3 ± 18.6	30.8 ± 5.1	250 mg/day phytosomal curcumin for 8 wk	37	43.1 ± 11.6	15	80.0 ± 11.9	29.2 ± 4.2	Placebo	5
4	Hariri 2020	23	40.95 ± 12.24	9	–	30.59 ± 5.91	250 mg/day phytosomal curcumin for 8 wk	22	40.06 ± 13.69	10	–	28.87 ± 3.59	Placebo	4
5	Cicero 2020	40	54 ± 3	22	–	27.1 ± 1.8	800 mg phytosomal curcumin for 8 wk	40	53 ± 5	21	–	26.9 ± 1.9	Placebo	5
6	Mirhafez 2019	32	45.45 ± 2.31	11	86.47 ± 3.59	30.55 ± 0.95	250 mg/day phytosomal curcumin for 8 wk	27	45.45 ± 2.31	11	86.47 ± 3.59	30.55 ± 0.95	Placebo	4
7	Panahi 2017	44	44.8 ± 11.14	14	83.86 ± 20.81	30.06 ± 5.76	1 000 mg/day phytosomal curcumin for 8 wk	43	40.7 ± 11.83	8	76.99 ± 16.68	27.72 ± 5.97	Placebo	4

Among the 7 eligible RCTs, 6 RCTs reported BMI,^[8,23–25,32,33]^ 2 RCTs reported body fat percent,^[8,25]^ 4 RCTs reported weight,^[8,23,25,32]^ 4 RCTs reported waist circumference,^[8,23,25,33]^ and 3 RCTs reported hip circumference and waist/hip ratio.^[8,24,25]^

### 3.2. Assessment of risk of bias

**Figure 2.**
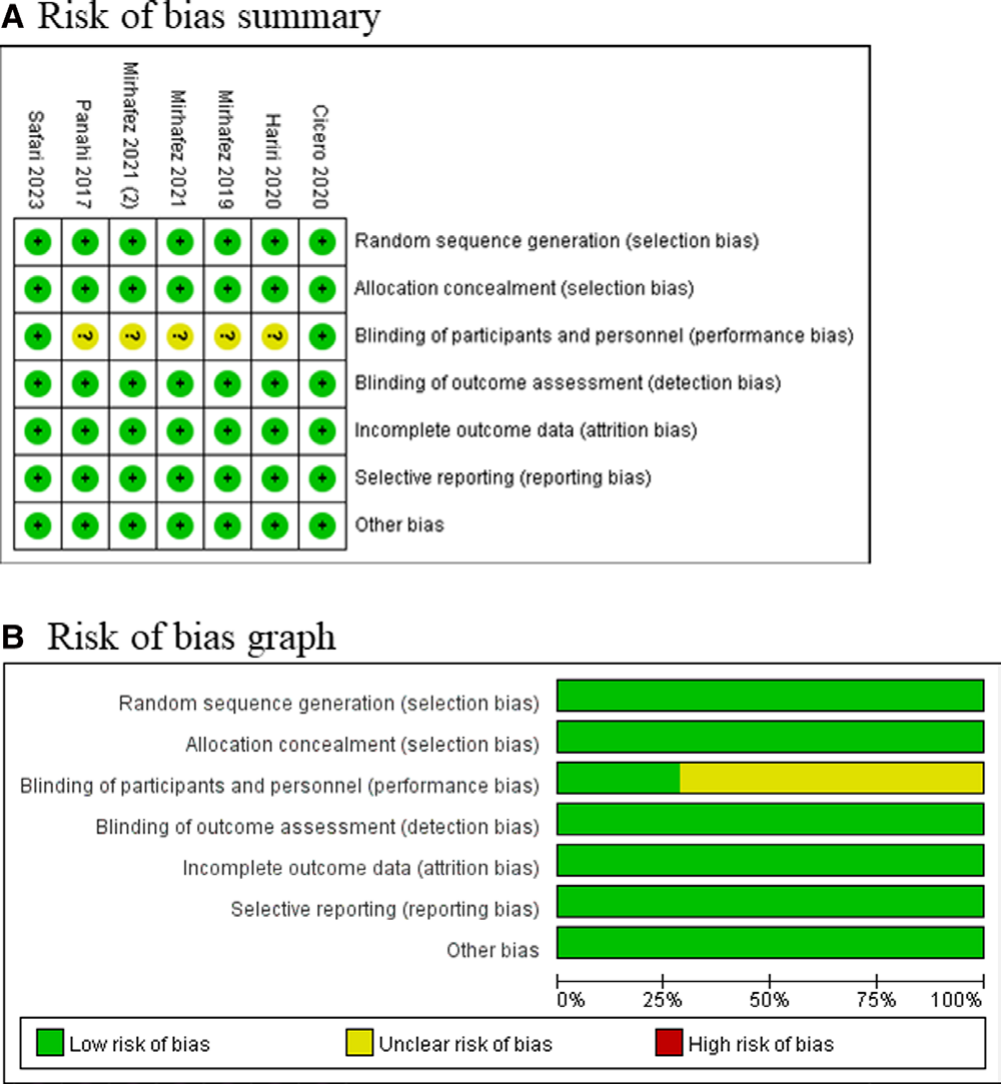
Risk of bias assessment. (A) Authors’ judgments about each risk of bias item for each included study. (B) Authors’ judgments about each risk of bias item presented as percentages across all included studies.

Risk of bias analysis (Fig. 2) showed that 5 studies had unclear risk of permeance bias due to unclear blinding,^[8,24,25,32,33]^ but all RCTs generally had high quality.

### 3.3. Primary outcomes: BMI and body fat percent

**Figure 3.**
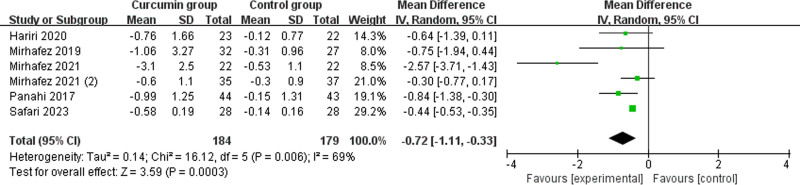
Forest plot for the meta-analysis of BMI (kg/m^2^). BMI = body mass index.

**Figure 4.**
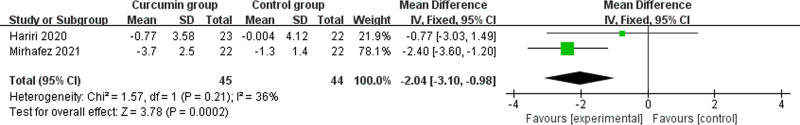
Forest plot for the meta-analysis of body fat percent.

BMI and body fat percent were very crucial for the control of nonalcoholic fatty liver disease. The results unraveled that compared to placebo in nonalcoholic fatty liver disease, phytosomal curcumin resulted in significantly reduced BMI (MD = −0.72; 95% CI = −1.11 to −0.33; *P* = .0003) with significant heterogeneity among the studies (*I*^2^ = 69%, heterogeneity *P* = .006, Fig. 3) and body fat percent (MD = −2.04; 95% CI = −3.10 to −0.98; *P* = .0002) with low heterogeneity among the studies (*I*^2^ = 36%, heterogeneity *P* = .21, Fig. 4).

### 3.4. Sensitivity analysis

Significant heterogeneity was seen for the BMI. As shown in Figure 2, the study conducted by Mirhafez et al^[8]^ showed results that were almost out of range of the others and probably contributed to the heterogeneity. After excluding this study, the results suggested that compared with placebo, phytosomal curcumin was still associated with lower BMI (MD = −0.45; 95% CI = −0.54 to −0.36; *P* < .00001), and no heterogeneity remained (*I*^2^ = 0, *P* = .57).

### 3.5. Secondary outcomes

**Figure 5.**
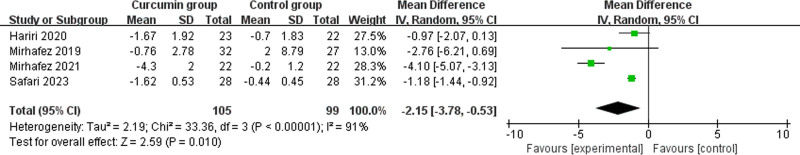
Forest plot for the meta-analysis of weight (kg).

**Figure 6.**
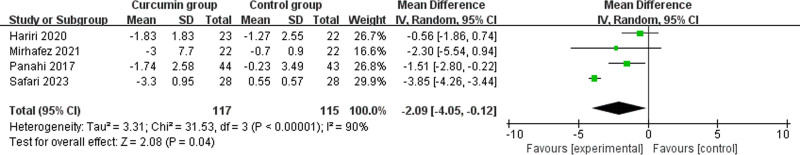
Forest plot for the meta-analysis of waist circumference (cm).

**Figure 7.**
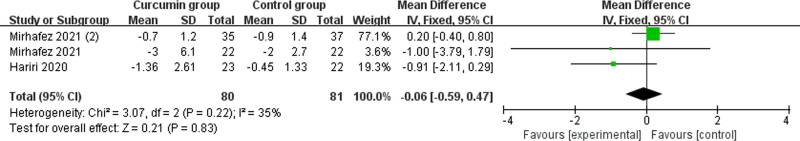
Forest plot for the meta-analysis of hip circumference (cm).

**Figure 8.**
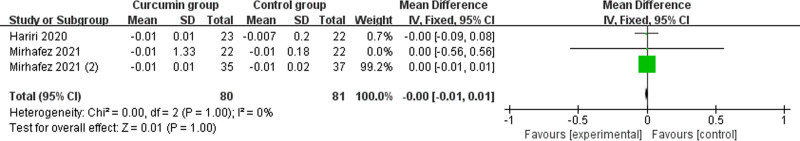
Forest plot for the meta-analysis of waist/hip ratio.

Other anthropometric indices such as weight, waist circumference, hip circumference and waist/hip ratio were also important for the efficacy assessment of phytosomal curcumin for nonalcoholic fatty liver disease. In comparison with placebo, phytosomal curcumin was associated with decreased weight (MD = −2.15; 95% CI = −3.78 to −0.53; *P* = .01; Fig. 5) and waist circumference (MD = −2.09; 95% CI = −4.05 to −0.12; *P* = .04; Fig. 6), but demonstrated no effect on hip circumference (MD = −0.06; 95% CI = −0.59 to 0.47; *P* = .83; Fig. 7) or waist/hip ratio (MD = 0; 95% CI = −0.01 to 0.01; *P* = 1; Fig. 8).

## 4. Discussion

In order to study the effect of phytosomal curcumin on anthropometric indices in patients with nonalcoholic fatty liver disease, our meta-analysis included 7 RCTs and 443 patients. The results confirmed that compared to control intervention, phytosomal curcumin could significantly reduce BMI, body fat percent, weight and waist circumference, but demonstrated no effect on hip circumference or waist/hip ratio. These implied that phytosomal curcumin was effective to improve anthropometric indices for nonalcoholic fatty liver disease.

Regarding the sensitivity analysis, there was significant heterogeneity for BMI. After excluding the study conducted by Mirhafez et al,^[8]^ no heterogeneity was seen. Several factors may result in this heterogeneity. Firstly, the treatment duration of phytosomal curcumin were different among the included RCTs, ranging from 8 weeks to 12 weeks. Secondly, patients with various duration and severity of nonalcoholic fatty liver disease may cause some bias. Thirdly, the doses of phytosomal curcumin ranged from 250 to 1000 mg daily.

One recent meta-analysis explored the influence of turmeric/curcumin on body weight, body mass index and waist circumference in nonalcoholic fatty liver disease. The results found no benefits of turmeric/curcumin supplementation on these anthropometric indices.^[34]^ In contrast, our results confirmed the efficacy of phytosomal curcumin to improve anthropometric indices for nonalcoholic fatty liver disease. These suggested that phytosomal curcumin had better efficacy for these patients than curcumin.

In terms of regulatory mechanisms, curcumin has important anti-oxidative properties that may reduce advanced glycation end-products and DNA damage among patients with nonalcoholic fatty liver disease.^[35,36]^ The antioxidant and anti-inflammatory properties of curcumin benefit to neutralize free radicals, and inhibit the production of prostaglandins and proinflammatory cytokines.^[37,38]^ In addition, curcumin showed the favorable effect on reducing blood pressure^[39]^ and liver enzymes for nonalcoholic fatty liver disease,^[40]^ as well as improving serum adiponectin concentration^[41]^ and oxidative stress in healthy females with moderate physical activity.^[42]^

We also should consider several limitations. Firstly, our analysis only included 7 RCTs, and more RCTs with large sample size should be conducted to confirm our findings. Secondly, there was significant heterogeneity during the sensitivity analysis, which may be caused by different doses and treatment durations of phytosomal curcumin. For instance, the consumption of 250 mg/day curcumin showed no improvement in liver enzyme levels,^[23]^ while curcumin in the doses above 1000 mg/day exerted benficial effect on liver enzymes.^[40]^ These results may indicate that phytosomal curcumin is more effective with higher doses. Thirdly, various severity of nonalcoholic fatty liver disease may produce some bias. Future studies should assess the efficacy of phytosomal curcumin in these patients with various severity. Fourthly, the protocol of this meta-analysis was not preregistered.

## 5. Conclusion

Phytosomal curcumin is able to improve anthropometric indices for nonalcoholic fatty liver disease.

## Author contributions

**Conceptualization:** Nana Liu, Hongting Li.

**Data curation:** Hongting Li.

**Formal analysis:** Nana Liu.

**Investigation:** Nana Liu.

**Methodology:** Hongting Li.

## Supplementary Material


